# Supplementary material for: Causal Relationship between Aging and Anorexia Nervosa: A White-Matter-Microstructure-Mediated Mendelian Randomization Analysis
Source: Biomedicines. 2024 Aug 16;12(8):1874. doi: 10.3390/biomedicines12081874 (PMC11351342; doi:10.3390/biomedicines12081874)
Supplement: Supplementary file 1 [file biomedicines-12-01874-s001.zip › Supplementary Figure/Figure S8/Leaveoneout plot of MR analysis for TL to ubm-b-1476.pdf]

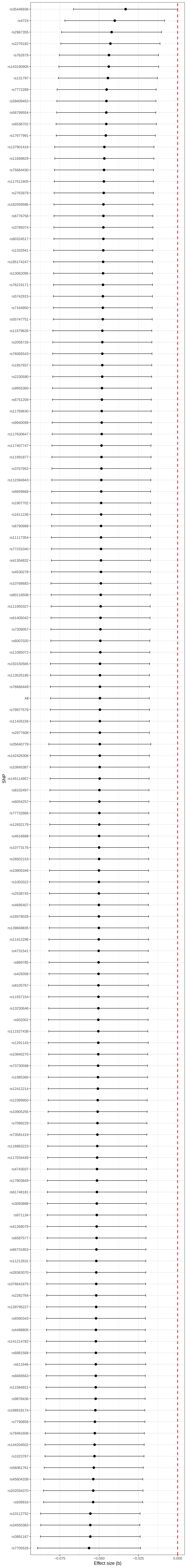

rs35446936
rs4724
rs2967355
rs2276182
rs762679
rs143190905
rs131797
rs7772289
rs59409453
rs56799554
rs6536702
rs17677991
rs137901416
rs11699829
rs75664430
rs117512405
rs2763979
rs182059586
rs6776756
rs3785074
rs80324517
rs1332941
rs185174247
rs13062095
rs76219171
rs5742915
rs7164950
rs55747751
rs11579626
rs2056726
rs76065543
rs1957937
rs2230590
rs9955360
rs6751209
rs11769630
rs9940099
rs117630647
rs117407747
rs11991877
rs3767952
rs112394943
rs6659669
rs1907702
rs1611236
rs6790988
rs11117354
rs77231040
rs41304832
rs4530278
rs10768683
rs80116508
rs111950327
rs61405042
rs7209057
rs6007020
rs11085072
rs150150565
rs113525195
rs76666449
All
rs79977579
rs11426156
rs2977608
rs35640778
rs142426306
rs10845387
rs145114957
rs8102497
rs6054257
rs77732866
rs12932179
rs4616688
rs10773176
rs28502153
rs10805346
rs1003322
rs2538745
rs4695407
rs16978028
rs139669835
rs11412296
rs4731541
rs869785
rs429358
rs8105767
rs11557154
rs13230646
rs932002
rs111527438
rs1291143
rs10840270
rs73730598
rs1985369
rs12412214
rs12369950
rs10905255
rs7099229
rs73581419
rs116863223
rs117034449
rs4743037
rs17803849
rs61748181
rs3093888
rs871134
rs41269079
rs6587577
rs66731853
rs11212631
rs28363070
rs376641875
rs2282764
rs139795227
rs6590343
rs4498805
rs141214782
rs6881568
rs611646
rs6669563
rs11584821
rs9878436
rs188918174
rs7790856
rs78491606
rs144204502
rs1023767
rs56061761
rs45604339
rs202034370
rs939916
rs10112752
rs34550383
rs3891167
rs7705526
SNP
−0.075
−0.050
−0.025
0.000
Effect size (b)
